# A Cancelable Iris- and Steganography-Based User Authentication System for the Internet of Things

**DOI:** 10.3390/s19132985

**Published:** 2019-07-06

**Authors:** Wencheng Yang, Song Wang, Jiankun Hu, Ahmed Ibrahim, Guanglou Zheng, Marcelo Jose Macedo, Michael N. Johnstone, Craig Valli

**Affiliations:** 1 Security Research Institute, Edith Cowan University, Perth, WA 6207, Australia; 2 Department of Engineering, La Trobe University, Melbourne, VIC 3083, Australia; 3 School of Engineering and Information Technology, University of New South Wales, Canberra, ACT 2600, Australia

**Keywords:** iris, feature data protection, cancelable, steganography

## Abstract

Remote user authentication for Internet of Things (IoT) devices is critical to IoT security, as it helps prevent unauthorized access to IoT networks. Biometrics is an appealing authentication technique due to its advantages over traditional password-based authentication. However, the protection of biometric data itself is also important, as original biometric data cannot be replaced or reissued if compromised. In this paper, we propose a cancelable iris- and steganography-based user authentication system to provide user authentication and secure the original iris data. Most of the existing cancelable iris biometric systems need a user-specific key to guide feature transformation, e.g., permutation or random projection, which is also known as key-dependent transformation. One issue associated with key-dependent transformations is that if the user-specific key is compromised, some useful information can be leaked and exploited by adversaries to restore the original iris feature data. To mitigate this risk, the proposed scheme enhances system security by integrating an effective information-hiding technique—steganography. By concealing the user-specific key, the threat of key exposure-related attacks, e.g., attacks via record multiplicity, can be defused, thus heightening the overall system security and complementing the protection offered by cancelable biometric techniques.

## 1. Introduction

In Internet of Things (IoT) networks, things, also called smart objects, are connected by wireless networks, producing and consuming data in order to perform their function. The term, IoT, was proposed by Ashton [1] in 1999. Since then, the IoT has drawn increasing attention from researchers in both academia and industry [1]. Smart objects in the IoT are commonly bound with sensors and computing capabilities, which enable them to sense the surrounding environment, communicate with each other, and potentially make a decision without (or with limited) human intervention.

Because of the energy and computing constraints of smart objects (e.g., cameras), rather than relying on their limited resources, data need to be collected and transmitted wirelessly by smart objects to remote central servers for further processing in the scenario of remote surveillance IoT networks. However, for such IoT networks, security threats such as unauthorized access can significantly impact on data confidentiality and user privacy. Therefore, user authentication for the purpose of access control plays a key role in establishing trust between users of smart objects and remote servers. A reliable authentication system ensures that the users of smart objects are the genuine, legitimate users, so that trust can be established and data integrity can be guaranteed. The capability of an authentication system to detect imposters determines the trust level in the IoT environment [2].

Passwords and tokens are traditional methods of user authentication. However, passwords can be easily forgotten and tokens may be stolen or lost. As an alternative, biometric authentication is becoming more attractive since biometric traits cannot be lost and do not need to be remembered [3,4]. Biometric recognition systems achieve authentication based on “who you are”, because any individual’s biometrics, e.g., iris and fingerprints, are unique [2]. Many biometric traits can be used for biometric recognition, such as fingerprints, face, iris, and palm prints. Among these options, the iris is highly reliable due to the unique and stable features it offers [4]. The iris begins to form from the third month of embryonic life. The unique pattern on the iris’ surface is generated during the first year of life and formation of this unique pattern is random and unaffected by genes [5], so even identical twins have different iris patterns.

**Figure 1:**
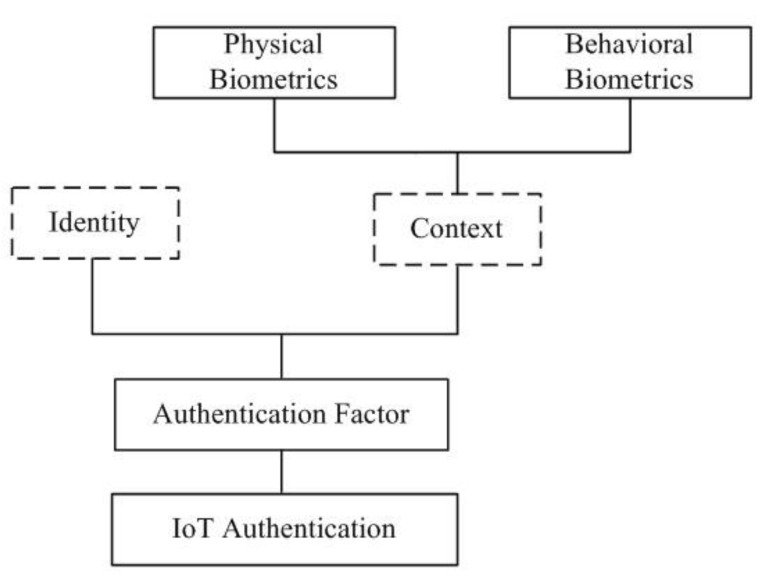
A partial taxonomy of authentication schemes for IoT devices (adapted from [6]).

IoT devices tend not to use source authentication for a range of reasons, which might be related to, for example, architectural constraints, power consumption, device memory, or simply the assumption that all devices in a network are trustworthy by default, and, therefore, authentication is unnecessary. El-hajj et al. [6] presented a survey of IoT authentication schemes. The authors pointed out that traditional authentication is unsuitable for the IoT setting due to the nature of IoT devices, despite ZigBee (as an example of a wireless IoT protocol) using 128 bit AES encryption. El-Hajj et al. [6] split authentication schemes according to the authentication factor, token use, authentication procedure, authentication architecture, IoT layer, and, finally, whether the scheme is hardware-based. In their analysis, biometric-based authentication has particular strengths, such as ease of use and unforgettable credentials, as well as resistance to certain types of attack. Figure 1 shows that biometrics methods fit into the physical context of IoT authentication.

With the aforementioned benefits of biometric authentication, one option is to leverage several biometrics in sequence in multi-modal verification, as reported by Blasco and Peris-Lopez [7]. Such a strategy may be better than non-biometric methods, but it relies on multiple biometrics, which is not necessarily feasible in the context of IoT devices. Nonetheless, the concept of combining authentication methods is sound, as noted by Arjona et al. [8], who used a combination of a biometric approach and a physically unclonable function. It is, therefore, worthwhile to consider fusing biometric recognition with another technique as a more secure means of authentication. Along this line of thinking, in this paper, we propose a cancelable iris- and steganography-based user authentication system for IoT networks. In the proposed scheme, the cancelable iris-based authentication system employs a user-specific secret key as the transformation parameter to guide non-invertible transformation. However, there is a potential risk associated with the user-specific key. That is, if it is acquired by an adversary, he/she may use it to restore the original iris feature data, which is likely to compromise the authentication system. To mitigate this potential risk, we integrate an effective information-hiding technique—steganography with cancelable iris biometrics. Unlike existing cancelable biometric authentication systems, in our scheme, the user-specific key is not generated and transmitted together with the users’ biometric data to the server for authentication purposes. Instead, it is hidden within other media data, e.g., collected images, which are sent to the server separately. Concealing the existence of the user-specific key enhances the security of the iris-based authentication system.

The rest of this paper is organized in the following order. Relevant research in the biometric-based IoT and cancelable iris-based biometrics are presented in Section 2. The cancelable iris- and steganography-based user authentication system is proposed in Section 3. In Section 4, experimental results are reported and discussed. Finally, the conclusion is provided in Section 5.

## 2. Related Work

### 2.1. Biometric-Based IoT Networks

With the advantages (e.g., uniqueness, convenience) of biometrics over password- and token-based traditional authentication, many researchers have been working on developing biometric-based methods for user authentication in IoT networks. For instance, in [2], Kashif et al. proposed an authentication framework using biometrics and wireless device radio fingerprinting for user authentication. The proposed framework not only can verify the monitored healthy data from the correct patient, but also ensures the integrity of the data. In [9], Kantarci et al. introduced a cloud-centric biometric identification architecture, which couples both the biometric scheme and context-aware technique to protect mobile applications from unauthorized access. In [10], Karimian et al. applied electrocardiogram (ECG) signals to authentication in an IoT system, as they observed that ECG biometrics are reliable, secure, and easy to implement. In [11], Maček et al. presented a scheme with multimodal biometrics (face and iris) for authentication. In this scheme, the face and iris images are obtained simultaneously using the high-quality, built-in cameras of mobile devices, e.g., laptops, smartphones, and tablets. One drawback of this scheme, as pointed out by the authors, is the acceptability of iris biometrics and the privacy concerns surrounding the stored face and iris templates.

In [12], Shahim et al. attempted to authenticate users using both the users’ hand geometry scan and a series of gestures on a Raspberry Pi platform. In [13], a lightweight multi-factor remote user authentication scheme was developed by Dhillon and Kalra. In the proposed scheme, the use of computationally less expensive hash functions and XOR (exclusive or) operations make the scheme suitable for resource-constrained IoT devices. In [14], Punithavathi et al. proposed a cloud-based lightweight cancelable fingerprint authentication system. The experimental results and analysis showed that the proposed fingerprint authentication system achieved state-of-the-art recognition performance with less computing time, thus rendering it a good candidate for IoT networks.

### 2.2. Cancelable Iris-Based Biometrics

Although the benefits of biometrics make biometric systems an appealing alternative to password- or token-based authentication for IoT devices, a major issue in biometric-based authentication systems is that any individual’s biometric traits are not replaceable. The loss of original biometric feature data in one application means that it is lost forever and also affects all other applications that use the same feature set [15,16]. The compromise of original biometric feature data leads to serious security and privacy concerns. Therefore, it is vital to protect the original biometric feature data. One important biometric data protection technique is known as cancelable biometrics. In a cancelable biometric system, the original biometric feature data are converted into an irreversible version by applying a one-way transformation. The transformed feature data are mathematically non-invertible, and, if compromised, they can be easily revoked and replaced with another transformed version by changing the parameter key, which is user-specific [17]. Cancelable biometrics was first proposed by Ratha et al. [18]. Later, three different transformation functions, Cartesian transformation, Polar transformation, and Functional transformation, were developed by Ratha et al. to generate a practical cancelable fingerprint authentication system [19].

Compared with those common biometric traits, e.g., fingerprints and face, the iris provides good reliability and high recognition accuracy, so it has been employed in many biometric authentication systems. There is ongoing research into cancelable iris biometrics. In [20], Zuo et al. proposed four different methods to generate cancelable iris biometrics for improving the security and privacy of iris templates. The authors also discussed the strengths and drawbacks of these four methods. In [21], Hämmerle-Uhl et al. introduced two different transformations, block re-mapping and mesh-warping. With different parameter settings, system performance can be well maintained with only marginal post-transformation degradation. For example, the block re-mapping achieved an equal error rate (EER) of 1.2% after transformation, compared with EER = 1.1% before transformation. In [22], Kanade et al. incorporated two factors, iris and password, to generate cancelable iris templates. Specifically, a user-specific key is used to shuffle the iris code and an Error Correcting Code (ECC) is employed to decrease feature variation to achieve better recognition performance. In [23], Pillai et al. designed cancelable iris biometrics based on sectored random projections. Two steps, feature extraction and random projections, are included in this method. The experimental results show that the criterion for cancelability is met.

In [24], Jenisch and Uhl applied block permutation and remapping to protecting the iris template. Specifically, in the permutation operation, blocks of the feature texture are rearranged, controlled by a permutation key, and in the remapping operation, some blocks are mapped on top of the other blocks to make the reconstruction of the iris image more difficult. In [25], Hämmerle-Uhl et al. implemented key-dependent wavelet transformations to build non-invertible iris templates. In this approach, the extracted iris features are highly sensitive to slight variations in key parameters. The experimental results show that the accuracy of the proposed scheme is similar before and after feature transformation.

In [26], Rathgeb et al. presented an adaptive Bloom filter-based cancelable iris recognition system. The Bloom filters can map part of a binary template to Bloom filter-based representations, which are irreversible. This system is alignment-free because the Bloom filter-based features do not require image pre-alignment. In [4], Lai et al. introduced the “Indexing-First-One” (IFO) hashing. Two mechanisms, Hadamard product code and module thresholding functions, are proposed to further improve the security and performance of the IFO hashing.

The existing issue: In the abovementioned iris-based authentication schemes, the non-invertible transformations rely on user-specific keys (or parameters), which can also be referred to as key-dependent transformations. However, in some schemes, e.g., [21,24], when the key, which is used to guide the transformation, is known by an adversary, the transformation can be reversed easily. In random projection based schemes, e.g., [23], if multiple transformed feature vectors and keys are lost, the adversary can restore the original feature vector from the attacks via record multiplicity (ARM) [27,28]. The exposure of the user-specific key leaks useful information, which may be exploited by the adversary. In this case, the security of the iris recognition system is under threat. In order to enhance key protection, Tran et al. [29] split the key into different camouflage images based on Shamir’s Secret Sharing Scheme. However, this approach is cloud-based and does not suit the conventional operation under discussion.

Contributions of this work: To reduce the risk introduced by the exposure of user-specific keys, we propose a cancelable iris- and steganography-based user authentication system. This system uses a cancelable biometric technique to secure the original biometric data. Furthermore, the steganography [30] technique is employed to hide the user-specific key, which is required by cancelable biometrics. In this way, we can enhance the overall security of the user authentication system by complementing the protection provided by a cancelable biometric technique.

## 3. The Proposed Cancelable Iris- and Steganography-Based System

**Figure 2:**
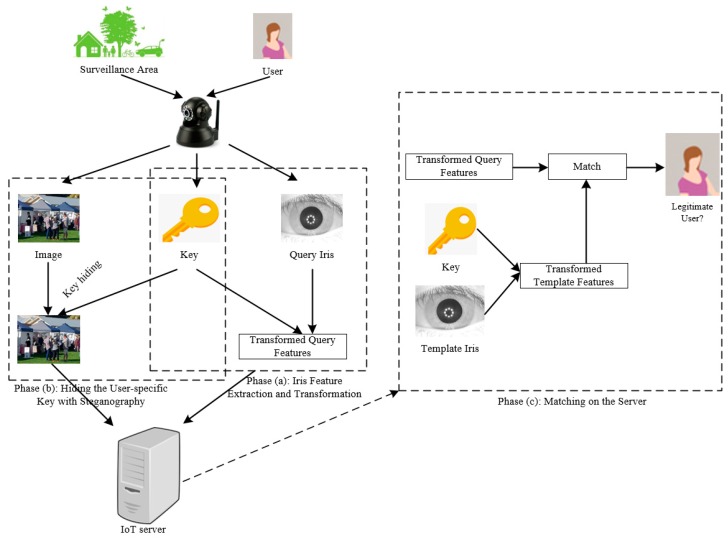
The entire authentication process of the proposed system.

In a remote surveillance IoT network, smart objects are responsible for continuously monitoring targeted areas and transmitting monitored data, (e.g., images) back to the server. In certain cases, the user may need to access a smart object for an update. To prevent unauthorized access, user authentication plays a critical role. In this paper, user authentication is performed by a cancelable iris- and steganography-based authentication system. The entire user authentication process of the proposed system is illustrated in Figure 2, which includes three major phases—Phase (a): iris feature generation and transformation, Phase (b): hiding the user-specific key with steganography, and Phase (c): matching on the server. These three phases correspond to Section 3.1, Section 3.2, and Section 3.3, respectively. 

To focus on the relevant issues, we assume that the user of smart objects has registered his/her iris (i.e., template data stored in the server) prior to the deployment of the IoT network. The server has superior computing power and security compared to the deployed smart objects. The following processes (Section 3.1, Section 3.2 and Section 3.3) are carried out when a user wants to access the IoT devices or services through the proposed user authentication system. 

### 3.1. Iris Feature Extraction and Transformation

**Figure 3:**
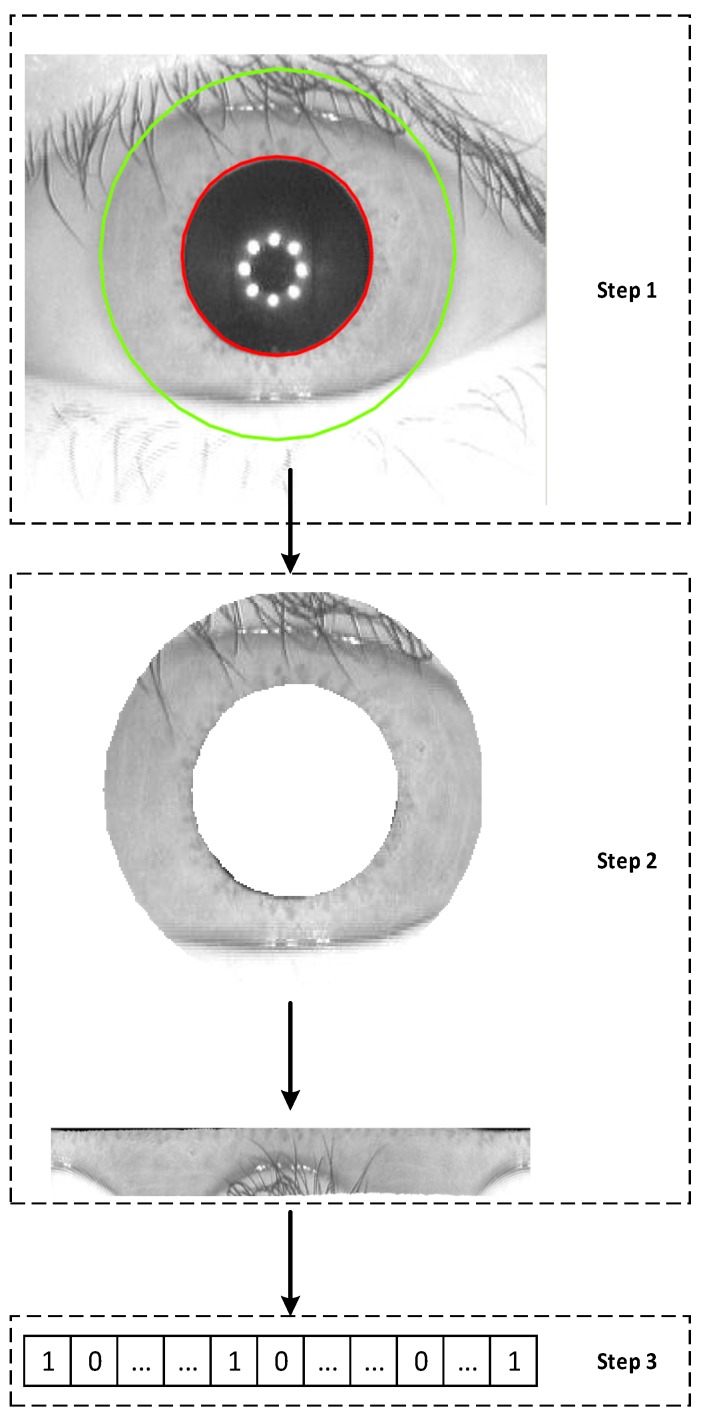
Three iris feature generation steps—Step 1: segmentation, Step 2: normalization, and Step 3: feature extraction.

When a camera captures an image of a user’s iris, three steps are typically required for the authentication system to generate features from the iris image, as demonstrated in Figure 3. The first step is to isolate the iris region, which is called iris segmentation. The iris region is defined as the area between two circles, one circle being the boundary between the iris and sclera (the green circle in Step 1 of Figure 3) and the other circle being the boundary between the iris and pupil (the red circle in Step 1 of Figure 3). After the iris region is isolated and segmented from the eye image, the second step is normalization, which unwraps the iris region into a rectangle with fixed dimensions, as shown in Step 2 of Figure 3. With the normalization step, two eye images of the same iris under different conditions can provide features at the same spatial location. The last step is feature extraction, as shown in Step 3 of Figure 3. This step creates the iris feature vectors in a specific data format, e.g., a binary string.

There are many existing algorithms for extracting iris features from an iris image, such as Masek’s algorithm [5] and the algorithm in [31]. In this paper, VeriEye SDK (Software Development Kit) [32] from Neuro Technology is adopted to extract iris features. Assume that F is the original iris feature extracted by VeriEye SDK. This feature vector contains 2348 bytes, each of which is an integer in the range of [0, 255]. To reduce intra-class variation and also convert the integer values into binary, in this work, quantization is applied to each element of the feature vector F. Specifically, elements that are located in [0, 255 × 1/4] are represented by the binary value of ‘00’. Elements that fall in [255 × 1/4, 255 × 2/4] are assigned the binary value of ‘01’. Similarly, elements that belong to [255 × 2/4, 255 × 3/4] are given the binary value of ‘10’ and elements in the range of [255 × 3/4, 255] are represented by the binary value of ‘11’. After quantization, the original feature vector F is converted to a binary feature vector $F_{b}$. The size of $F_{b}$ is 4696 (= 2348 × 2) bits, because each element in F is quantized into two bits in $F_{b}$.

Because of iris rotation in the iris image acquisition process, after the binary feature vector $F_{b}$ is obtained, feature shifting is needed before further operations take place, such as non-invertible transformation and matching on the server. In this work, the template feature $F_{b}^{T}$ stored in the server does not need any shifting, but each query feature vector $F_{b}^{Q}$ is shifted up to *N* bits left and up to *N* bits right (the superscripts *T* and *Q* stand for ‘template’ and ‘query’). Each bit shift creates a new variant of the query feature vector $F_{b}^{Q}$. Therefore, including $F_{b}^{Q}$ itself, a total of 2*N* + 1 binary strings, $F_{B}^{Q}=\{F_{b}^{Q}(-N),\ldots ,F_{b}^{Q}(0),\ldots ,F_{b}^{Q}(N)\}$, are generated from this shifting operation, where “−” denotes left shift and $F_{b}^{Q}(0)$ means $F_{b}^{Q}$ itself without shifting (i.e., a 0 bit shift).

If the query feature set $F_{B}^{Q}$ is directly sent to the server without any protection and obtained by the adversary, the original iris features can be retrieved, leading to serious consequences, such as identity loss. To protect $F_{B}^{Q}$, we employ a random projection based transformation, guided by a user-specific key [33]. Specifically, whenever the proposed authentication system receives a user’s iris image, a new user-specific key K is generated as a seed to construct a projection matrix M, which is of size m×n, where m≤n. Then, the non-invertible transformation is applied to each element of the query feature set $F_{B}^{Q}$, given by

(1) $$y^{Q}(i)=MF_{b}^{Q}(i)$$

where i∈[−N,N]. As a result, a feature set containing 2*N*+1 vectors, $Y^{Q}={\left\{y^{Q}(i)\right\}}_{i\in [-N,N]}$ is generated. The application of random projection in Equation (1) forms an underdetermined system that has non-unique solutions. Even if both the transformed feature vector $y^{Q}(i)$ and the projection matrix M (or user-specific key K) are obtained by the adversary [34], it is computationally hard to find the query vector $F_{b}^{Q}(i)$.

### 3.2. Hiding the User-Specific Key with Steganography

The transformed query feature vector $y^{Q}(i)$ is derived from Equation (1) using the projection matrix M generated by the user-specific key K as a seed. If $K_{1}$ is set to be different to $K_{2}$, the generated projection matrix, $M_{1}$, is different to $M_{2}$. The security of $F_{b}^{Q}(i)$ in Equation (1) is based on a well-known result about an underdetermined system of linear equations. However, according to [27,28], if the adversary can acquire multiple transformed feature vectors and their corresponding projection matrixes (or user-specific key K), then the original query feature vector $F_{b}^{Q}(i)$ can be retrieved by launching the ARM (attacks via record multiplicity). Therefore, protecting the secret key K is critical in defending against the ARM.

To protect the user-specific key K, we chose an established information-hiding technique named steganography to hide K in a cover image [30]. It is noteworthy that steganography differs from cryptographic techniques. A cryptographic method would scramble the key so that it cannot be understood, while steganography hides the key so it cannot be seen. There are some popular methods in steganography. For example, see e.g., [35,36], where different redundancies in a cover image are exploited for hiding data. In [37], the data is hidden in the least significant bits (LSBs) of a cover image, and in [38], the data is hidden in the frequency domain.

**Figure 4:**
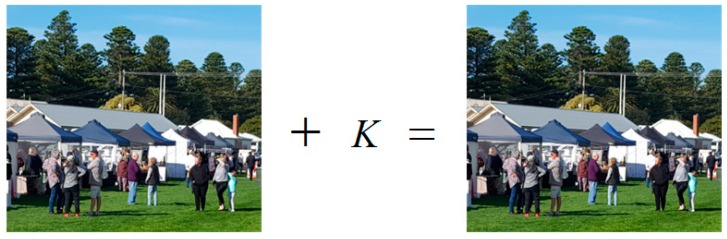
An image before key hiding (**left**) and after key hiding (**right**). The difference between them is impossible to distinguish with the naked eye.

Since the objective of this paper is to design an authentication system that improves the security of the cancelable iris biometrics by hiding the secret key K, we implemented an online steganography program [39]. An image before key hiding and after key hiding is shown in Figure 4, which is impossible to distinguish with the naked eye. Note that the cover image can be any image out of a number of images collected by smart objects in a targeted surveillance area.

### 3.3. Matching on the Server

After the operations of query feature transformation and key hiding with steganography, the transformed feature set $Y^{Q}$ is sent to the server, while the user-specific key K hidden in one of the numerous images is also sent to the server, but separately. Once the user-specific key K is retrieved at the server, the same transformation is performed to the stored template feature $F_{b}^{T}$, guided by the projection matrix M, which is generated by the user-specific key K. That is,

(2) $$y^{T}=MF_{b}^{T}.$$

In the matching process, the template feature vector $y^{T}$ is compared with each element in the query feature set $Y^{Q}={\left\{y^{Q}(i)\right\}}_{i\in [-N,N]}$. The similarity score between $y^{T}$ and each element $y^{Q}(i)$ in $Y^{Q}$ is calculated by using Equation (3) below:

(3) $$S_{i}=1-\frac{||y^{T}-y^{Q}(i)|{|}_{2}}{||y^{T}|{|}_{2}+||y^{Q}(i)|{|}_{2}}$$

where $||\cdot |{|}_{2}$ is the 2-norm. Then, a score array $S=[S_{0},S_{1},\ldots ,S_{2N}]$ is obtained and the maximum score $S_{\max}$ in S is chosen as the final matching score between the template and query iris images to reach a verdict. The similarity score $S_{\max}$ ranges from 0 to 1 with 1 meaning that the template and the query match exactly [40,41]. If the matching score is larger than a predefined threshold, then the query is a legitimate user registered in the server, and vice versa.

## 4. Experimental Results

### 4.1. Database Selection and Experimental Environment

The evaluation of the proposed method is conducted over the following three publicly available iris databases:

CASIA-IrisV3-Interval [42]: This database includes 2639 iris images from 395 classes (eyes) captured with a close-up iris camera. The resolution of the iris image is 320 × 280 pixels. In our experiments, we only selected the left eye images (a total of 1332 images).

MMU-V1 [43]: This database includes 450 images (five images per iris and two irises per subject), contributed by 45 individuals using a semi-automated camera, LG IrisAccess 2200, dedicated to Iris capturing. The resolution of the iris image is 320 × 240 pixels. All the images were involved in our experiments.

UBIRIS-V1-Session 1 [44]: This database contains 1214 iris samples from 241 individuals. The resolution of the iris image is 200 × 150 pixels. In our experiments, the first five iris samples of each of the 241 individuals from the first session were used (a total of 1205 images).

**Figure 5:**
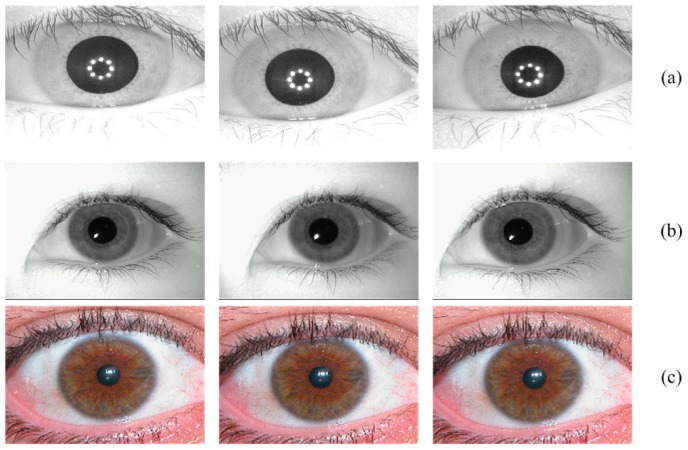
Three iris samples from the CASIA-IrisV3-Interval, MMU-V1, and UBIRIS-V1-Session 1 databases, in (**a**), (**b**), and (**c**), respectively.

Three samples from each database are illustrated as examples in Figure 5. The experiments in this work were conducted using MATLAB on a laptop with a 2.50 GHz Intel i5-2450M dual-core CPU, 8 GB of RAM, and a 64 bit Windows 7 operating system. Further, as noted in Section 3, the VeriEye SDK [32] from Neuro Technology was adopted to extract the iris features. Because the feature extraction of 179 images and 30 images from the CASIA-IrisV3-Interval and UBIRIS-V1-Session 1 databases was unsuccessful using VeriEye, they were excluded from the experiments.

### 4.2. Performance Evaluation

Three performance indicators were employed to measure system performance. They are (1) false rejection rate (FRR), (2) false acceptance rate (FAR), and (3) equal error rate (EER) [45]. In our experiments, the first image of each eye was considered as the template and the remaining images of the same eye were taken as the query to calculate the FRR, while the first image of each eye was regarded as the template and the first image of all other eyes was used as the query to calculate the FAR. The third indicator, EER, is defined as the error rate when FRR is equal to FAR.

The effect of shifting by a different number of bits was evaluated using original binary features (features before applying transformation), in order to find the optimal parameter setting of *N*. Note that because the original feature is in a binary format, we use Equation (4) from [46] to calculate the similarity score. Equation (3) in this paper is used to calculate the similarity score of the transformed feature data. The EERs of the proposed system using a different *N* with the original binary features on three different databases are listed in Table 1. It can be seen that, the system with the original binary features achieves the best performance when *N* = 4, 8, and 2 for the CASIA-IrisV3-Interval, MMU-V1, and UBIRIS-V1-Session 1 databases, respectively. Thus, these databases are chosen as the parameters for evaluating the system performance in the transformed domain.

#### 4.2.1. The Effect of Transformation Parameters on System Performance

**Figure 6:**
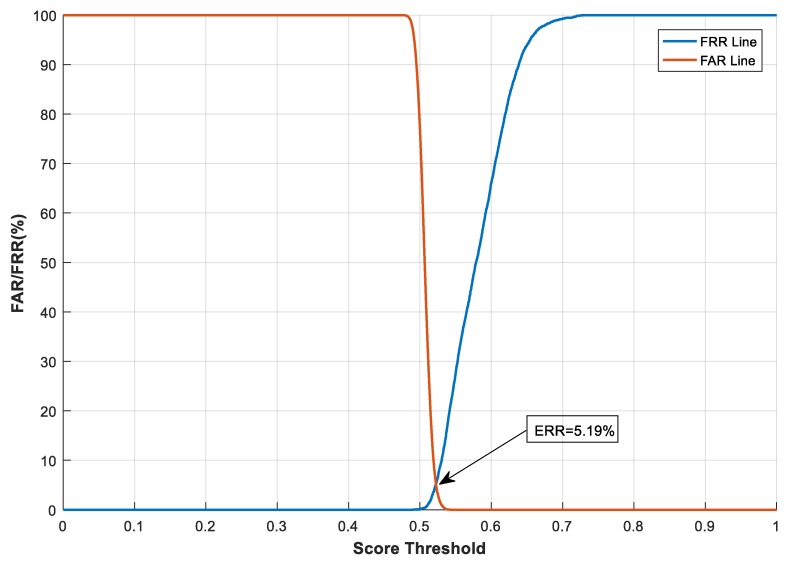
System performance under different score thresholds, with *m* = 500 on the CASIA-IrisV3-Interval database. FRR, false rejection rate; FAR, false acceptance rate.

**Figure 7:**
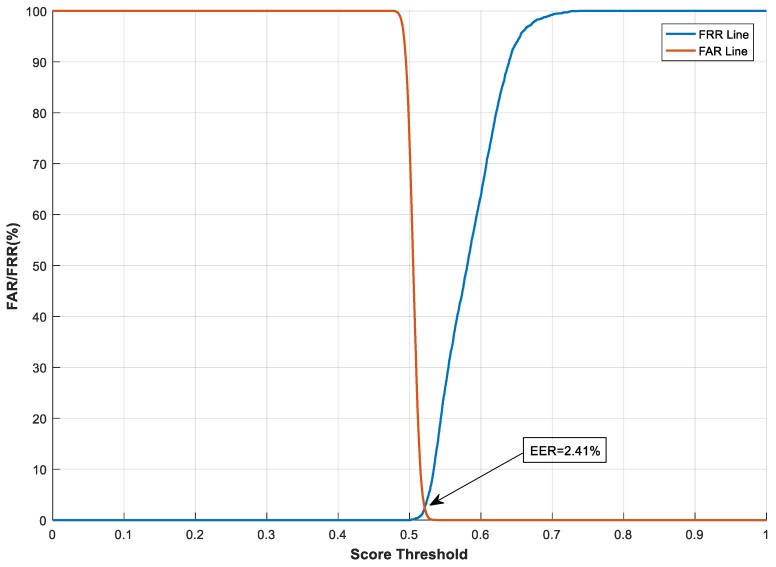
System performance under different score thresholds, with *m* = 1000 on the CASIA-IrisV3-Interval database.

**Figure 8:**
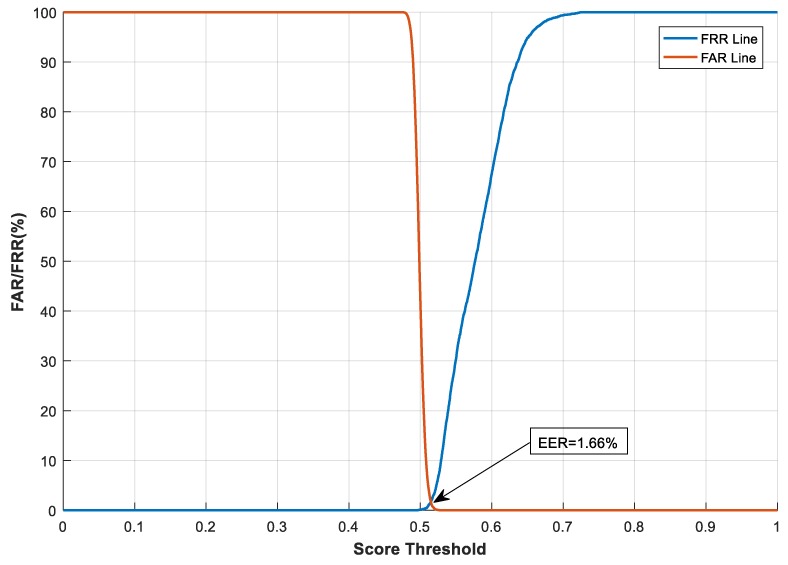
System performance under different score thresholds, with *m* = 2000 on the CASIA-IrisV3-Interval database.

**Figure 9:**
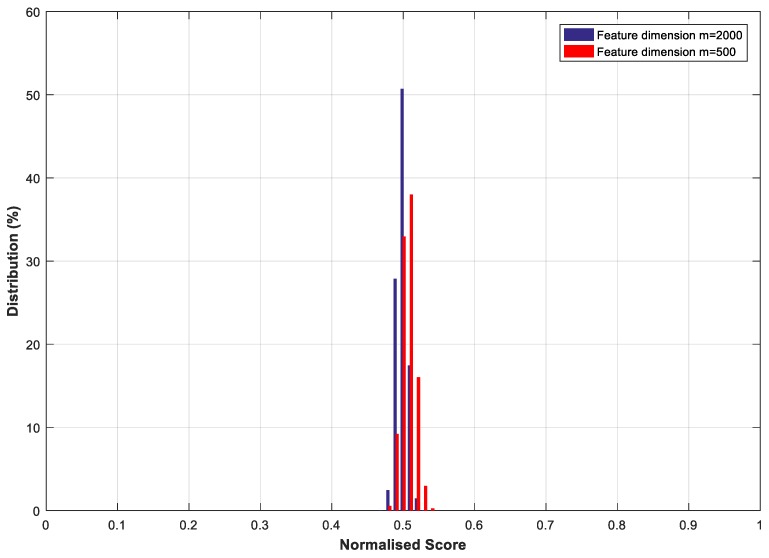
Distribution of the similarity scores of imposter tests with different feature dimensions on the CASIA-IrisV3-Interval database.

With the feature transformation, we also evaluated and analysed how the transformation parameters, e.g., the size (m×n) of the projection matrix M, impact system performance. This test was carried out on the CASIA-IrisV3-Interval database. Here, *n* has a fixed value of 4696, which is equal to the length of the binary feature vector $F_{b}$. We varied the value of *m* from 500 to 2000 to examine the effect of different sizes of the projection matrix on system performance. The Receiver Operating Characteristic (ROC) curves in terms of FAR and FRR [47] under different *m* values are shown in Figure 6, Figure 7 and Figure 8. In the figures, the similarity score threshold varies from 0 to 1. The performance of the proposed method under different *m* values is EER = 1.66%, 2.41%, and 5.19% when *m* = 2000, 1000, and 500, respectively. It can be seen that the proposed system performs worse as m decreases. This is because less information about the original features is preserved with a greater dimension cut (smaller *m*), leading to performance degradation. Moreover, similar to the analysis in [48], we evaluated the imposter score distribution of the proposed system using the transformed feature vector of different dimensions (i.e., giving *m* different values*)* on the CASIA-IrisV3-Interval database, as demonstrated in Figure 9. The mean and standard deviation of the similarity score distribution with dimension *m* = 2000 are 0.4988 and 0.0072, respectively, compared with 0.5070 (mean) and 0.0093 (standard deviation) when dimension *m* = 500. It can be seen that the differences in the mean and standard deviation values are very small—only 0.0082 and 0.0021, respectively. Although the difference in the feature dimensions of the two imposter tests causes such discrepancies, it also demonstrates that there is a safe and fairly constant dissimilarity distance when different transformed feature vectors are compared, according to [48].

#### 4.2.2. Comparison with Other Similar Systems

In the meantime, the performance of the proposed scheme is compared with other similar existing cancelable iris schemes, as shown in Table 2. It can be seen that experiments of most existing methods were carried out on the CASIA-IrisV3-Interval database, for which the proposed system performs better than [20,49], but slightly worse than other schemes. On the MMU-V1 and UBIRIS-V1-Session 1 databases, the proposed system outperforms the methods in [50,51]. More importantly, one obvious advantage of the proposed system is its heightened security, since the user-specific key K is hidden using steganography, which significantly increases the difficulty of launching key exposure-related attacks, e.g., ARM.

## 5. Conclusions

In this paper, we have designed a user authentication system for IoT networks. The proposed system is equipped with a cancelable iris and a steganography-based mechanism for key hiding. To protect the original iris data, feature quantization and shifting are conducted on the original feature vectors before the random projection-based feature transformation in order to achieve better recognition performance. Furthermore, to address the key exposure-related attacks, e.g., ARM, which existing key-dependent cancelable biometric systems are susceptible to, we propose to further enhance the security of the cancelable iris biometrics using steganography by hiding user-specific keys. In the future, we will investigate different types of transformation functions and study how to properly hide the secret key under various scenarios, e.g., in a mobile environment.

## Figures and Tables

**Table 1:** System performance with untransformed features using different parameter *N*. EER, equal error rate.

Shifting Parameter	*N* = 2	*N* = 4	*N* = 8
**CASIA-IrisV3-Interval**	EER = 0.62%	EER = 0.22%	EER = 0.22%
**MMU-V1**	EER = 2.11%	EER = 1.89%	EER = 1.77%
**UBIRIS-V1-Session 1**	EER = 2.43%	EER = 2.52%	EER = 2.53%

**Table 2:** System performance in terms of EER (%) using transformed features in comparison with similar methods.

Methods	Databases		
	CASIA-IrisV3-Interval	MMU-V1	UBIRIS-V1-Session 1
Bin-combo in Zuo et al. [20]	4.41%	-	-
Jenisch and Uhl [24]	1.22%	-	-
Uhl et al. [25]	1.07%	-	-
Rathgeb et al. [26]	1.54%	-	-
Ouda et al. [49]	6.27%	-	-
Jin et al. [4]	0.54%	-	-
Radman et al. [51]	-	-	9.48%
Zhao et al. [50]	1.06%	5.50%	13.44%
Proposed (*m* = 2000)	1.66%	4.78%	3.00%

EER of [20,49] are quoted from [4].
